# American Association of Obstetricians and Gynecologists

**Published:** 1890-11

**Authors:** 


### AMERICAN ASSOCIATION OF OBSTETRICIANS AND GYNECOLOGISTS.

From *The Canadian Practitioner.*

The annual meeting of the American Association of Obstetricians and Gynecologists took place in Philadelphia, on the 16th, 17th, and 18th of September, 1890. This society has for its object the discussion of the kindred subjects of obstetrics, diseases of women, and abdominal surgery. The first portion of the programme was confined to obstetrical subjects, and the remainder to gynecology and abdominal surgery, in relation to the new advances in the treatment of obstruction of the ureter, intestinal diseases, diseases of the gall bladder, and peritonitis. The chair was taken by the president, Dr. E. E. Montgomery, of Philadelphia. The very efficient secretary, Dr. W. W. Potter, of Buffalo, was in his place, and to his faultless arrangements much of the success of the meeting was due. Everything proceeded with clock-like regularity. Among those present, memory serves to mention the following: Drs. E. E. Montgomery, J. Price, Baldy, and Hoffman, Philadelphia; Dr. W. W. Potter, Buffalo; Dr. Rohé, Baltimore; Drs. Vander Veer and Townsend, Albany; Drs. A. H. Wright and J. F. W. Ross, Toronto; Drs. Reed and Hall, Cincinnati; Dr. Davis, Birmingham, Ala.; Dr. Seymour, Troy, N. Y.; Dr. Werder, Pittsburg, Pa.; and others.

The discussions on many important matters connected with abdominal surgery were especially valuable. Dr. Vander Veer, with commendable courage, read reports of some very interesting cases, on which he invited criticism. The paper was not, as many papers are, a record of easy successes or of difficult cases with successful recoveries, but a record, faithfully prepared, of cases operated upon as a last resort after many delays, some with fatal termination, some with successful termination, and some with partially successful termination. One case recorded, of artificially-established intestinal anastomosis, is an addition to the records of the history of intestinal surgery that should encourage other workers in the same field. The patient died subsequently, but the intestines were found completely united, although a contraction had occurred at the artificial opening. If any one is sceptical as to the kind of union that will occur between the ends of resected intestine, he has but to visit the Army Medical Museum in Washington, and examine the specimen presented by Dr. Halstead, now of Johns Hopkins

Hospital. Union is so complete that the scar cannot be discovered except by most careful examination.

Another paper of particular interest was that presented by Dr. Hall, of Cincinnati, on removal of an impacted calculus from the ureter by combined abdominal and lumbar incision. The stone, after giving rise to symptoms of paroxysmal pain for three years, was discovered after making an exploratory incision, and was removed through secondary incision in the loin. The point of greatest interest was the cause of the pain. The stone was bean-shaped, and at the hilum a small slit existed through which, when clear and free from mucus, the urine trickled, but through which the urine could not find its way if blocked. This blocked condition produced a distention of the ureter and pelvis above the obstruction, and gave rise to the pain.

The specimens shown by Dr. Joseph Price were of very great interest. Myomata sloughing, as a result of electrolytic puncture, were shown. Dr. Price showed many specimens and plates of pyosalpinx, matted ovaries and tubes, from patients in whose pelves electricity had been given a fair trial by the most skilled electrical operators. He thought that if these electricians knew more of gynecology and less of electricity, the patients would be better off. A veil of mystery had been thrown around the use of electricity, and before a man was looked upon as fit to use it he must spend half a lifetime in his preliminary education. The general consensus of opinion was that the results of electrical treatment were disappointing, and that the cases came into the hands of the abdominal surgeon eventually; that electricity is a good caustic; that it will temporarily relieve hemorrhage in some cases of uterine fibroids; that it is useless in tubal disease or disease of the ovaries, or in ectopic gestation. As to its power of relieving pelvic pain, not much was said. Many reported results of the use of electricity were useless, owing to the impossibility of making a correct diagnosis without opening the abdomen. A supposititious diagnosis may be made, but it is not and cannot be verified without exploratory operation. To exemplify this point, Dr. Price showed a specimen removed before many of the members in the forenoon. He thought the woman had had a miscarriage and was suffering from puerperal pyosalpinx. He would defy any member present to diagnosticate the case even now with the specimen in his hand. Was it pyosalpinx or was it tubal pregnancy? He then opened it with a scalpel and it proved to be a tubal pregnancy. The fetus was easily made out.

He held that Tait's idea of rupture into the broad ligament was not correct. He had seen many cases (and the one just shown proved the fact) in which no rupture had taken place into the broad ligament, but the tube had simply gone on dilating as it does in pyosalpinx, hematosalpinx, and hydrosalpinx.

A discussion of the supposed virtues of the Staffordshire knot took place, a case having been mentioned in which hemorrhage had taken place after the most careful use of the knot. Dr. Price said that he had long since given up using it, and had returned to the simple old knot used by many celebrated ovariotomists of the past. The simple transfixion, crossing of the ligatures, and tying in halves, would not allow bleeding any more than any other kind of knot. It was not so much the knot as the man who tied it.

There can be no doubt that hemorrhage may occur after using any kind of knot, no matter how well applied. All of the best operators have had this experience.

The question of drainage after abdominal operations was discussed. Some thought drainage should be used in every case; others that it should be used very seldom. The general weight of opinion seemed to be that drainage should be used whenever the abdomen was washed out, where there was danger of subsequent hemorrhage from adhesions, or a thick pedicle where there was much handling of the peritoneum.

The question of the necessity of drainage after washing out a tuberculous or an inflamed peritoneum was not discussed.

The wonderful record of Dr. Joseph Price, of twenty-six consecutive abdominal hysterectomies without a death, was presented to the meeting in an informal way.

The question of the advisability of performing vaginal hysterectomy for cancer was discussed, and opinion still remains divided. The statistics on both sides have not yet been collated to the satisfaction of those holding neutral ground. Many are waiting to be convinced. The main questions seem to be: 1. Do the cases live longer after (*a*) vaginal hysterectomy, (*b*) after high or low amputation of the cervix, or (*c*) after being left practically alone, or occasionally cauterized to relieve the hemorrhage?

2. Is the suffering greater up to the time of death (*a*) if they are simply occasionally cauterized; (*b*) if the disease returns after vaginal hysterectomy; or (*c*) if the disease returns after amputation of the cervix?

3. In how many cases can we be sure (*a*) that if the uterus is removed, that surrounding structures are not already affected ; (*b*) that if the cervix is removed, we have not already a nodule of disease in the fundus ?

These points require consideration, and were brought up in the discussion, but were not satisfactorily answered owing to the limited data from which to draw conclusions. If these questions could be answered by the collated cases of Tait, who is opposed to any operation ; of Leopold, the ardent advocate of the operation ; and of Van de Warker, the advocate of high amputation, and discussed by these surgeons at an international congress, a conclusion might be arrived at satisfactory to all concerned.

The treatment advised for gunshot wounds or stabs of the abdomen was immediate exploration by abdominal section. One case was cited in which a box-car at a railway siding was used as an operating room, the damaged intestines were immediately repaired, and the boy made an excellent recovery.

Dr. M. Price related a remarkable case of gunshot wound. The ball traversed the liver, injured the intestines, and went through the kidney. He repaired the intestines, resected the liver, and, with the exception of a small abscess in the kidney, the girl made a good recovery.

Dr. E. E. Montgomery, in presenting a specimen of extremely early ectopic gestation removed by operation, took occasion to state that he had altered the views expressed by him at a previous meeting regarding the advisability of using electricity during the early months of ectopic gestation. He knew from subsequent experience that it was a useless agent in such cases.

The social features of the meeting were very enjoyable. Dr. E. E. Montgomery received the Fellows at his residence on the evening of Tuesday, the 16th. A cordial invitation was extended to the Fellows to be present at a reception given by Dr. Willard, at the Art Club, to the Fellows of the Orthopedic Association. On Wednesday evening, a sumptuous banquet was given to the Fellows by the Obstetrical Society of Philadelphia. A most enjoyable evening was spent. Dr. Parish presided.

On Thursday, the newly-elected president, Dr. Adam H. Wright, of Toronto, took the chair, and, after a few appropriate remarks, adjourned the meeting *sine die.* The members returned home, to the north, south, east, and west, to gather another harvest for the next meeting, a year hence.

The place of meeting has not yet been decided upon, but I feel sure that if the executive committee could be induced to select Toronto, the profession at large would make their visit so pleasant that they would be willing to return to us at no distant date.

Looking critically over the proceedings, one might justly say that, while a few of the papers were not up to the average, most of them were of a high order, and evoked discussions equal to any discussions I have ever heard, either in the American Gynecological Society, British Gynecological Society, or those we have read from the transactions of other special societies. We were particularly struck with the results obtained in abdominal surgery in all parts of this continent. Western, northern, and southern people need go no longer to New York to have their abdomens opened, because they can have them operated upon as well, if not better, with every comfort, and without incurring so much expense, much nearer home.

J. F. W. R.

